# *Maioricimonas rarisocia* gen. nov., sp. nov., a novel planctomycete isolated from marine sediments close to Mallorca Island

**DOI:** 10.1007/s10482-020-01436-z

**Published:** 2020-06-25

**Authors:** Elena Rivas-Marin, Sandra Wiegand, Nicolai Kallscheuer, Mareike Jogler, Stijn H. Peeters, Anja Heuer, Mike S. M. Jetten, Christian Boedeker, Manfred Rohde, Damien P. Devos, Christian Jogler

**Affiliations:** 1 Centro Andaluz de Biología del Desarrollo, CSIC, Universidad Pablo de Olavide, Seville, Spain; 2 Institute for Biological Interfaces 5, Karlsruhe Institute of Technology, Eggenstein-Leopoldshafen, Germany; 3 Department of Microbiology, Radboud Universiteit, Nijmegen, The Netherlands; 4 Department of Microbial Interactions, Friedrich-Schiller University, Jena, Germany; 5 Leibniz Institute DSMZ, Brunswick, Germany; 6 Central Facility for Microscopy, Helmholtz Centre for Infection Research, Brunswick, Germany

**Keywords:** Marine bacteria, Planctomycetes, PVC superphylum, Mallorca coast, *Planctomicrobium piriforme*, *Thalassoglobus*

## Abstract

Planctomycetes are ubiquitous bacteria with environmental and biotechnological relevance. Axenic cultures of planctomycetal strains are the basis to analyse their unusual biology and largely uncharacterised metabolism in more detail. Here, we describe strain Mal4^T^ isolated from marine sediments close to Palma de Mallorca, Spain. Strain Mal4^T^ displays common planctomycetal features, such as division by polar budding and the presence of fimbriae and crateriform structures on the cell surface. Cell growth was observed at ranges of 10–39 °C (optimum at 31 °C) and pH 6.5–9.0 (optimum at 7.5). The novel strain shows as pear-shaped cells of 2.0 ± 0.2 × 1.4 ± 0.1 µm and is one of the rare examples of orange colony-forming Planctomycetes. Its genome has a size of 7.7 Mb with a G+C content of 63.4%. Phylogenetically, we conclude that strain Mal4^T^ (= DSM 100296^T ^= LMG 29133^T^) is the type strain representing the type species of a novel genus, for which we propose the name *Maioricimonas rarisocia* gen. nov., sp. nov.

## Introduction

Planctomycetes are bacteria that belong to the PVC superphylum (Wagner and Horn 2006), which includes the phyla *Planctomycetes*, *Verrucomicrobia, Chlamydiae, Lentisphaerae* and *Kirimatiellaeota* as well as some uncultured candidate phyla, such as *Candidatus* Omnitrophica. The PVC superphylum has environmental, medical and biotechnological relevance (Devos and Ward 2014).

Planctomycetes have been shown to be present in several environments, in which they play important roles in biogeochemical cycles, such as the carbon and nitrogen cycle (Wiegand et al. 2018). One example are Planctomycetes of the class *Candidatus* Brocadiae, which perform unique reactions during anaerobic ammonium oxidation (anammox) (Strous et al. 1999; Peeters and van Niftrik 2019). Members of the phylum *Planctomycetes,* in particular of the class *Planctomycetia*, colonise a variety of environments from terrestrial to aquatic, being able to dwell on various marine algal surfaces (Bengtsson et al. 2012; Bondoso et al. 2014, 2015, 2017; Lage and Bondoso 2014; Vollmers et al. 2017). They form biofilms on biotic surfaces (Bengtsson and Øvreås 2010), on which they metabolise complex carbon substrates (Lachnit et al. 2013; Jeske et al. 2013). Unique pili-forming crateriform structures and an enlarged periplasm are probably required for uptake and also cleavage of large polysaccharides obtained from the environment (Boedeker et al. 2017).

Planctomycetes possess large genomes with sizes of up to 12.4 Mb (Ravin et al. 2018), in which the presence of giant genes has been reported (Jeske et al. 2013; Guo et al. 2014; Kohn et al. 2016; Faria et al. 2018). These genome sizes are in line with their assumed capacity for secondary metabolite production (Graça et al. 2016; Jeske et al. 2016; Yadav et al. 2018). Furthermore, several members of the phylum *Planctomycetes* produce carotenoids, which could be associated with an increased tolerance against UV radiation or oxidative stress (Kallscheuer et al. 2019b).

Planctomycetes were considered exceptional due to several presumptively eukaryotic features, such as the lack of a peptidoglycan (König et al. 1984), a compartmentalised cell plan (Lindsay et al. 1997), a nucleus-like structure (Fuerst and Webb 1991) and the endocytosis-like uptake of macromolecules for an intracellular degradation (Lonhienne et al. 2010). However, with advances of microscopy techniques and the development of genetic tools (Jogler et al. 2011; Rivas-Marín et al. 2016b; Boedeker et al. 2017), many of these traits have been refuted or reinterpreted.

In recent years, the presence of peptidoglycan has been reported in several members of the *Planctomycetes* (Jeske et al. 2015; van Teeseling et al. 2015) and also in the sister phyla *Verrucomicrobia* (Rast et al. 2017) and *Chlamydiae* (Pilhofer et al. 2013; Liechti et al. 2014, 2016). With the exception of anammox-performing Planctomycetes (Jogler 2014; Neumann et al. 2014), the proposed cell plan has been found to feature large invaginations of the cytoplasmic membrane instead of closed compartments (Santarella-Mellwig et al. 2013; Acehan et al. 2014; Boedeker et al. 2017). These discoveries contributed to the reinterpretation of Planctomycetes as bacteria with a cell envelope architecture resembling that of Gram-negative bacteria, but with some variations (Devos 2014a, b; Boedeker et al. 2017). Nevertheless, Planctomycetes remain exceptional in other ways, e.g. they lack the protein FtsZ normally essential for bacterial division as well as other division proteins (Pilhofer et al. 2008; Jogler et al. 2012; Rivas-Marín et al. 2016a). Beyond that, phylum members divide by binary fission, budding or intermediate mechanisms (Wiegand et al. 2018, 2020). Presence and essentiality of sterols in the membranes of one of its members was recently reported (Pearson et al. 2003; Rivas-Marin et al. 2019).

The unusual cell biology of Planctomycetes prompted us to explore the uncharacterised planctomycetal diversity. In the present study, we describe the novel strain Mal4^T^ isolated from marine sediments in Palma de Mallorca (Spain) in terms of physiological, microscopic as well as genomic properties. Supported by phylogenetic analyses, we conclude that strain Mal4^T^ represents a novel species of a novel genus within the family *Planctomycetaceae*.

## Materials and methods

### Cultivation conditions and isolation

Strain Mal4^T^ was isolated from marine sediments at the coast of S’Arenal close to Palma de Mallorca (Spain) on the 23th of September 2014 (sampling location: 39.5126 N 2.7470 E) as previously described (Wiegand et al. 2020). For strain isolation and cultivation M1H NAG ASW medium was used. Medium preparation was previously described (Kallscheuer et al. 2019a). Cultures were incubated in baffled flasks at 28 °C with constant agitation at 110 rpm. Plates were cultivated at 28 °C for 2–3 weeks and isolated colonies were then streaked on fresh M1H NAG ASW plates. Initial amplification and sequencing of the 16S rRNA gene, intended to check whether isolated strains are members of the phylum *Planctomycetes,* was performed as previously described (Rast et al. 2017).

### Physiological analyses

Cultivations for physiological assays were performed in M1H NAG ASW medium. For pH optimum determination, 100 mM 2-(*N*-morpholino)ethanesulfonic acid (MES) was used for cultivations at pH 5.0, 5.5, 6.0 and 6.5. For cultivations at pH values ranging from 7.0 to 8.0, MES was replaced by 100 mM 4-(2-hydroxyethyl)-1-piperazine-ethanesulfonic acid (HEPES), whereas 100 mM 3-(4-(2-Hydroxyethyl)piperazin-1-yl)-propane-1-sulfonic acid (HEPPS) served as a buffering agent at pH 8.5 and 100 mM *N*-cyclohexyl-2-aminoethanesulfonic acid (CHES) was used for pH maintenance at pH 9.0 and 9.5. Cultivations for determination of the pH optimum were performed at 28 °C. For temperature optimum determination, strain Mal4^T^ was cultivated at pH 8.0 at different temperatures ranging from 10 to 39 °C. Cell growth and maximal growth rates were inferred from optical density measurements at 600 nm (OD_600_) of triplicate cultures.

### Genome analysis

The genome of strain Mal4^T^ was previously published (Wiegand et al. 2020). The genome (accession number CP036275) and 16S rRNA gene sequence (accession number MK559979) are available from the GenBank database. The primary metabolism was analysed by examining locally computed InterProScan (Mitchell et al. 2019) results cross-referenced with information from the UniProt database (UniProt 2019) and BlastP results of ‘typical’ protein sequences. Numbers of carbohydrate-active enzymes were determined by employing dbCAN2 (Zhang et al. 2018), which automatically mines the CAZy database (Lombard et al. 2014).

### Light microscopy and scanning electron microscopy

Phase contrast microscopy and scanning electron microscopy were performed according to protocols published earlier (Kallscheuer et al. 2019a).

### Phylogenetic analyses

16S rRNA gene sequence-based phylogeny was computed for strain Mal4^T^, the type strains of all described planctomycetal species (assessed in January 2020) and all isolates recently published (Kohn et al. 2016, 2020a, b; Boersma et al. 2019; Kallscheuer et al. 2019a; Dedysh et al. 2020; Wiegand et al. 2020). An alignment of 16S rRNA gene sequences was performed with SINA (Pruesse et al. 2012). The phylogenetic analysis was conducted employing a maximum likelihood approach with 1000 bootstraps, the nucleotide substitution model GTR, gamma distribution and estimation of proportion of invariable sites (GTRGAMMAI option) (Stamatakis 2014). Three 16S rRNA genes of bacterial strains from the PVC superphylum, but outside of the phylum *Planctomycetes*, were used as outgroup. The *rpoB* nucleotide sequences (encoding the RNA polymerase β-subunit) were taken from publicly available genome annotations and the sequence identities were determined as described previously (Bondoso et al. 2013) using Clustal Omega (Sievers et al. 2011). Alignment and matrix calculation were done after extracting only those parts of the sequence that would have been sequenced with the described primer set. The average nucleotide identity (ANI) was calculated using OrthoANI (Lee et al. 2016). The average amino acid identity (AAI) was obtained with the aai.rb script of the enveomics collection (Rodriguez-R and Konstantinidis 2016). The percentage of conserved proteins (POCP) was calculated as described before (Qin et al. 2014). The unique single-copy core genome of all analysed genomes for the multi-locus sequence analysis (MLSA) was determined with proteinortho5 (Lechner et al. 2011) (‘selfblast’ option enabled). The sequences of the obtained orthologous groups were aligned using MUSCLE v.3.8.31 (Edgar 2004). After clipping, partially aligned C- and N-terminal regions and poorly aligned internal regions were filtered using Gblocks (Castresana 2000). The final alignment was concatenated and clustered using the maximum likelihood method implemented by RaxML (Stamatakis 2014) with the ‘rapid bootstrap’ method and 500 bootstrap replicates.

## Results and discussion

### Phylogenetic inference

**Fig. 1:**
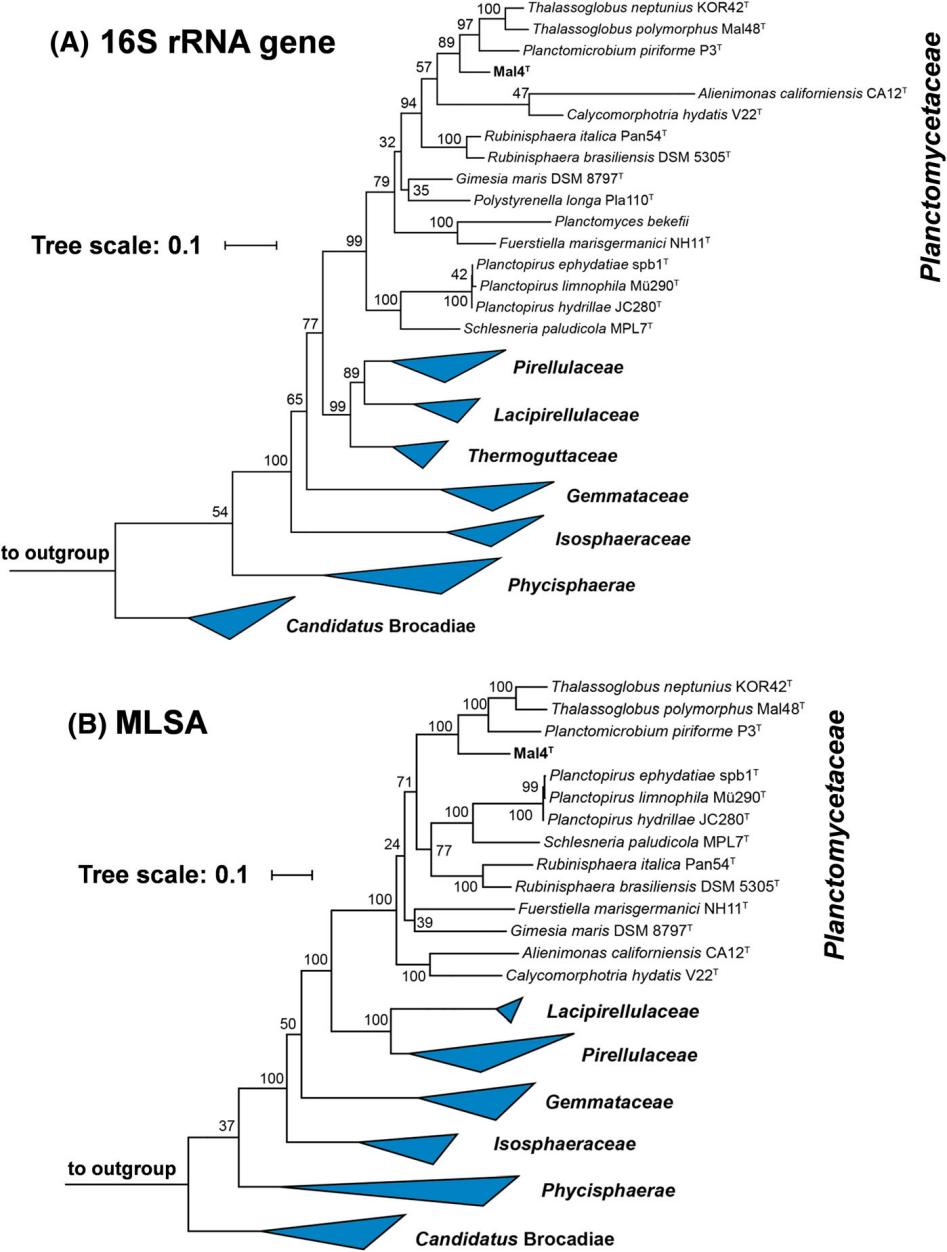
Phylogenetic trees highlighting the position of strain Mal4^T^. The outgroup consists of three 16S rRNA genes from the PVC superphylum outside of the phylum *Planctomycetes*. Bootstrap values from 1000 re-samplings (500 re-samplings for MLSA) are given at the nodes (in %)

**Fig. 2:**
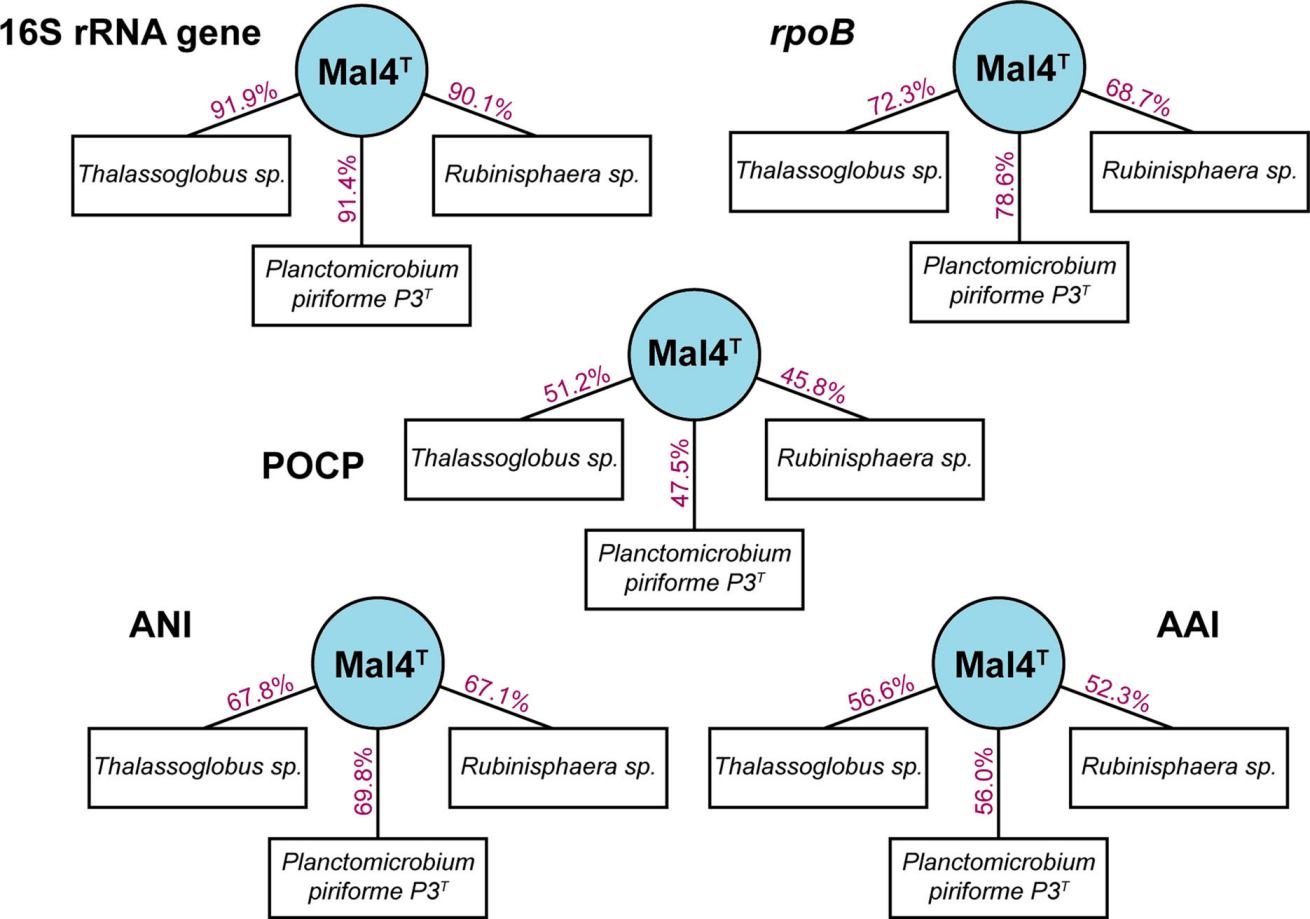
Similarity values of the novel isolate Mal4^T^ in relation to species *P. piriforme* P3^T^, *Thalassoglobus* sp. *and Rubinisphaera* sp. Methods used: 16S rRNA gene sequence identity, *rpoB* gene identity, percentage of conserved proteins (POCP), average nucleotide identity (ANI) and average amino acid identity (AAI)

In the phylogenetic trees obtained after analysis of 16S rRNA gene sequences, as well as MLSA (Fig. 1), strain Mal4^T^ clusters stably with members of two genera of the family *Planctomycetaceae*, namely *Planctomicrobium* and *Thalassoglobus*. 16S rRNA gene sequence identity between strain Mal4^T^ and the two genera is between 91.4% and 91.9% (Fig. 2). These values are below the proposed genus threshold of 94.5%, but above the threshold for separate families of 86.5% (Yarza et al. 2014), indicating that strain Mal4^T^ represents an distinct genus in the family *Planctomycetaceae*. Coherently, average nucleotide identities (ANI) below 95% confirm that strain Mal4^T^ is a distinct species. Phylogenetic assumptions on the genus level can also be obtained by analysing the *rpoB* gene sequence identities, AAI and POCP. For delineation of genera, the proposed threshold values for the above-mentioned markers are 75.5–78% (Kallscheuer et al. 2019c), 45–65% (Konstantinidis et al. 2017) and 50% (Qin et al. 2014), respectively. The *rpoB* identity value and the AAI between strain Mal4^T^ and the members of the genus *Thalassoglobus*, which comprises *Thalassoglobus neptunius* KOR42^T^ (Kohn et al. 2020a) and *Thalassoglobus polymorphus* Mal48^T^ (Rivas-Marin et al. 2020), are below the given thresholds. POCP was found to be slightly above the threshold (51.2%), this, however, does not significantly influence the overall conclusion that strain Mal4^T^ belongs to a separate genus. Minimal comparative values of strain Mal4^T^ and the genus *Rubinisphaera*, another closely related genus; featuring *Rubinisphaera italica* (Kallscheuer et al. 2019a) and *Rubinisphaera brasiliensis* (Scheuner et al. 2014), are below these thresholds for all three phylogenetic markers (Fig. 2). Analogously, POCP between strain Mal4^T^ and *Planctomicrobium piriforme* P3^T^ (Kulichevskaya et al. 2015) was also found to fall below the proposed threshold (Fig. 2), whilst the AAI value (56%) was in a ‘grey zone’ (45–65%), but well below the upper limit. Although the *rpoB* gene sequence identity of 78.6% is slightly above the proposed threshold, this sole deviance should not overrule the distinctiveness of the other values. In summary, the majority of analysed phylogenetic markers suggests that strain Mal4^T^ belongs to a novel genus.

### Morphological and physiological analyses

**Fig. 3:**
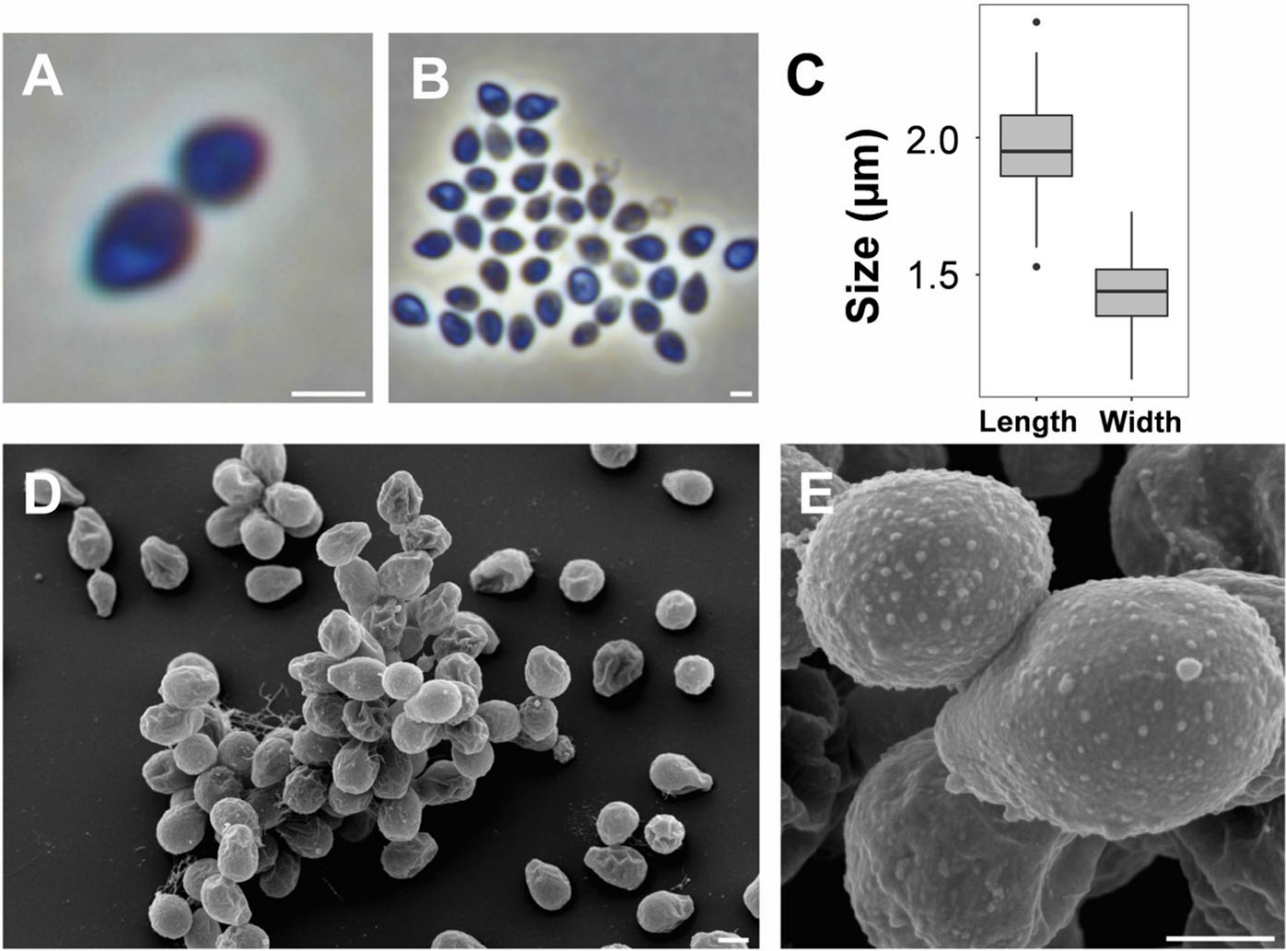
Microscopy images and cell size plot of strain Mal4^T^. Pictures from light microscopy (**a**, **b**) and scanning electron microscopy (**d**, **e**) are shown. The scale bars are 1 µm. For determination of the cell size (**c**) at least 100 representative cells were counted manually or by using a semi-automated object count tool

**Fig. 4:**
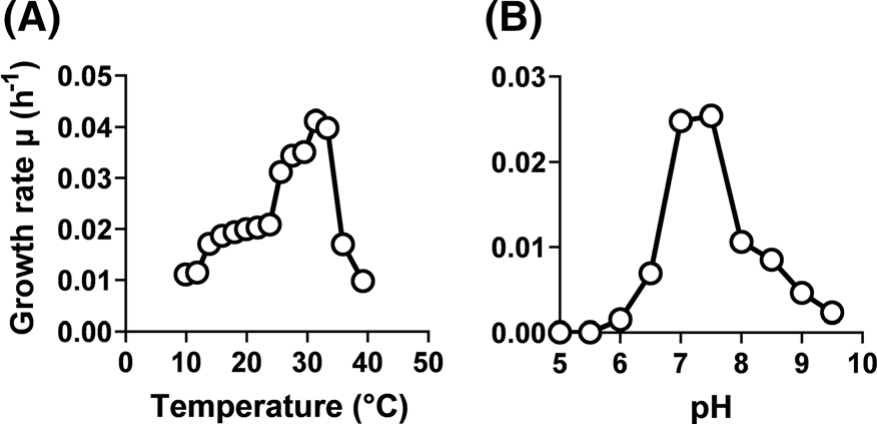
Temperature and pH optima of strain Mal4^T^. Data points show average growth rates obtained after cultivation in M1H NAG ASW medium in biological triplicates. Cultivations at different temperatures (**a**) were performed at pH 8. Cultivations at different pH values (**b**) were conducted at 28 °C

Light microscopy and scanning electron microscopy (Fig. 3) were applied to analyse the morphological characteristic of Mal4^T^ cells harvested during the exponential growth phase. Detailed information on morphology and cell division is summarised in comparison to the current closest relatives (Table 1). Mal4^T^ cells are pear-shaped (2.0 ± 0.2 µm × 1.4 ± 0.1 µm) (Fig. 3a–c), occur as single cells and in rare cases form aggregates (Fig. 3d). The cell surface appears rough, evenly covered with crateriform structures and short fimbriae (Fig. 3d, e). A holdfast structure was not observed during electron microscopy. As shown for all described members of the family *Planctomycetaceae*, cell division takes place by polar budding with the daughter cell displaying a round shape. Optimal temperature and pH for growth were shown to be 31 °C and pH 7.5, respectively, however, Mal4^T^ cells are able to grow over a range of 10–39 °C and pH 6.5–9.0 (Fig. 4). These values are comparable to the two *Thalassoglobus* species, but differ from *P. piriforme* P3^T^, which did not grow at temperatures exceeding 30 °C and favours moderate acidic conditions. The maximal observed growth rate of strain Mal4^T^ in M1H NAG ASW medium was determined to be 0.041 h^−1^, corresponding to a generation time of approximately 17 h. Strain Mal4^T^ is amongst the rare examples of planctomycetal strains forming orange colonies and might thus be an interesting strain for further analysis of carotenoid production and their function in Planctomycetes. Since most of the planctomycetal strains characterised so far are either pink to red or lack pigmentation (white), the pigmentation of the novel strain is an important phenotypic feature separating it from its current close phylogenetic neighbours. Strain Mal4^T^ is an aerobic heterotroph.

**Table 1:** Phenotypic and genotypic features of strain Mal4^T^ in comparison to its current closest relatives

Characteristics	Mal4^T^	*Thalassoglobus polymorphus* Mal48^T^	*Thalassoglobus neptunius* KOR42^T^	*Planctomicrobium piriforme* P3^T^*
Phenotypic features				
Color	Orange	Beige	Cream	White
Size (µm)	2.0 × 1.4	1.6 × 0.9	1.7 (diameter)	1.7–2.8 × 0.9–1.3 μm
Shape	Pear-shaped	Pear-shaped	Spherical	Ellipsoidal to pear-shaped
Temperature range (optimum) (°C)	10–39 (31)	15–36 (30)	22–36 (33)	10–30 (20–28)
pH range (optimum)	6.5–9.0 (7.5)	6.5–8.0 (7.5)	5.5–8.5 (7.0–7.5)	4.2–7.1 (6.0–6.5)
Aggregates	Yes	Yes	Yes	Yes
Division	Budding	Budding	Budding	Budding
Dimorphic life cycle	n.o.	n.o.	n.o.	Yes
Flagella	n.o.	n.o.	n.o.	Yes
Crateriform structures	Yes, overall	n.o.	Yes	Yes, polar
Fimbriae	Yes, overall matrix or fibre	Yes, overall matrix or fibre	Few fibres	Yes
Capsule	n.o.	n.o.	n.o.	n.d.
Bud shape	Round	Like mother cell	Round	Like mother cell
Budding pole	Polar	Polar	n.o.	Polar
Stalk	Yes	Yes	n.o.	Yes
Holdfast structure	n.o.	n.o.	Yes	Yes
Genotypic features				
Genome size (bp)	7,744,989	6,357,355	6,734,412	6,317,004
Plasmids (bp)	No	No	n.d.	n.o.
G+C content (%)	63.4	50.3	52.8	58.8
Protein-coding genes	5829	4874	5508	5050
Protein-coding genes/Mb	753	767	818	799
Hypothetical proteins	2257	1987	2516	2814
Coding density (%)	85.9	84.9	85.7	85.8
16S rRNA genes	2	2	1	1
tRNA genes	55	41	70	53

*Genomic data from GenBank acc. no. NZ_FOQD00000000

*n.o.* not observed, *n.d.* not determined

### Genomic characteristics

The genomic characteristics of strain Mal4^T^ in comparison to *T. polymorphus* Mal48^T^, *T. neptunius* KOR42^T^ and *P. piriforme* P3^T^ are summarised in Table 1. Its genome is 7.7 Mb in size, which is around 1 Mb larger compared to the other three strains. The G+C content is also the highest of the four strains. Automated gene prediction and annotation identified 5829 putative protein-encoding genes, of which 39% (2257 genes) are annotated as hypothetical proteins. These values correspond to 753 protein-coding genes per Mb and a coding density of 85.9%. Although the genome size of strain Mal4^T^ is larger, the coding density is in the same range in the other three species. Similar to its relatives, the strain lacks plasmids. Numbers of 41–55 tRNA genes are similar, except for *T. neptunius * KOR42^T^, which has a slightly higher number of 70 tRNA genes. As for *T. polymorphus* Mal48^T^, strain Mal4^T^ harbours two copies of the 16S rRNA gene, whereas the gene occurs in single copy in the other two strains.

### Genome-encoded features of the primary carbon metabolism

Based on the genome sequences, we analysed key metabolic capabilities in the primary metabolism of strain Mal4^T^ in comparison to the two *Thalassoglobus* species and *P. piriforme* P3^T^ (Table 2). Genes coding for enzymes participating in glycolytic pathways, gluconeogenesis, the tricarboxylic acid (TCA) cycle and anaplerotic reactions, such as pyruvate or phosphoenolpyruvate carboxylation and the glyoxylate shunt, were included. The resulting data suggest that strain Mal4^T^ is able to metabolise carbohydrates using at least two glycolytic pathways, the Embden-Meyerhof-Parnas pathway (the most common glycolytic pathway) and the pentose phosphate pathway. Additionally, its genome bears genes coding for putative 2-dehydro-3-deoxyphosphogluconate aldolase and phosphogluconate dehydratase, both involved in the alternative Entner-Doudoroff pathway. All four strains harbour a complete gene set required for a functional TCA cycle, which suggests that the central carbon metabolism of the strains is similar to canonical heterotrophic bacteria. With regard to gluconeogenesis, a minimal gene set required for this pathway has been identified, suggesting that the three strains are capable of *de novo* sugar biosynthesis. All four strains lack the glyoxylate shunt, which is typically required during growth either with acetate or with compounds that are degraded to acetate or acetyl-CoA units. The lack of the glyoxylate shunt suggests that the strains are not able to use such compounds as the exclusive energy and carbon source. Alternatively, they may follow a different pathway with a similar function.

**Table 2:** Genome-based primary metabolism of strain Mal4^T^ compared to the close relatives *Thalassoglobus polymorphus * Mal48^T^, *Thalassoglobus neptunius * KOR42^T^ and *Planctomicrobium piriforme* P3^T^

Enzyme	EC number	Gene	Mal4^T^	Mal48^T^	KOR42^T^	P3^T^*
Glycolysis (Embden–Meyerhof–Parnas pathway)						
Glucose-6-phosphate isomerase	5.3.1.9	*pgi*	Mal4_41440	Y	Y	Y
ATP-dependent 6-phosphofructokinase isozyme 1	2.7.1.11	*pfkA*	Mal4_28800	Y	Y	Y
Fructose-bisphosphate aldolase class 2	4.1.2.13	*fbaA*	Mal4_06980	Y	Y	Y
Triosephosphate isomerase	5.3.1.1	*tpiA*	Mal4_33520	Y	Y	Y
Glyceraldehyde-3-phosphate dehydrogenase	1.2.1.12	*gapA*	Mal4_10410	Y	Y	Y
Phosphoglycerate kinase	2.7.2.3	*pgk*	Mal4_43170	Y	Y	Y
2,3-Bisphosphoglycerate-independent phosphoglycerate mutase	5.4.2.12	*gpmI*	Mal4_57980	Y	Y	n.a.
2,3-Bisphosphoglycerate-dependent phosphoglycerate mutase	5.4.2.11	*gpmA*	N	N	N	Y
Enolase	4.2.1.11	*eno*	Mal4_26950	Y	Y	Y
Pyruvate kinase I	2.7.1.40	*pykF*	Mal4_16440	N	N	Y
Pyruvate dehydrogenase complex	1.2.4.1/2.3.1.12	*aceEF*	Mal4_36650/ Mal4_31770	Y	Y	Y
Gluconeogenesis						
Phosphoenolpyruvate synthase	2.7.9.2	*ppsA*	N	N	N	n.a.
Pyruvate, phosphate dikinase	2.7.9.1	*ppdK*	Mal4_37310	Y	Y	Y
Pyruvate carboxylase	6.4.1.1	*pyc*	Mal4_02890	Y	Y	Y
Phosphoenolpyruvate carboxykinase (ATP)	4.1.1.49	*pckA*	Mal4_29720	N	N	Y
Phosphoenolpyruvate carboxykinase (GTP)	4.1.1.32	*pckG*	N	N	N	N
Phosphoenolpyruvate carboxykinase (diphosphate)	4.1.1.38	*PEPCK*	Mal4_57980	Y	Y	n.a.
Fructose-1,6-bisphosphatase class 2	3.1.3.11	*glpX*	N	N	N	n.a.
Fructose-1,6-bisphosphatase class 1	3.1.3.11	*fbp*	Mal4_30740	N	N	n.a.
Pyrophosphate–fructose 6-phosphate 1-phosphotransferase	2.7.1.90	*pfp*	Mal4_26620	Y	Y	Y
Pentose phosphate pathway						
Glucose-6-phosphate 1-dehydrogenase	1.1.1.49	*zwf*	Mal4_45260	Y	Y	Y
6-Phosphogluconolactonase	3.1.1.31	*pgl*	Mal4_13550, Mal4_58700, Mal4_20230	Y	Y	Y
6-Phosphogluconate dehydrogenase, decarboxylating	1.1.1.44	*gndA*	Mal4_26580	Y	Y	Y
Transketolase 2	2.2.1.1	*tktB*	Mal4_57590/Mal4_57600	Y	Y	Y
Transaldolase B	2.2.1.2	*talB*	Mal4_01090	Y	Y	Y
KDPG (Entner–Doudoroff pathway)						
KDPG aldolase	4.1.2.14	*eda*	Mal4_43780	Y	Y	Y
Phosphogluconate dehydratase	4.2.1.12	*edd*	Mal4_05000	Y	Y	Y
TCA cycle						
Citrate synthase	2.3.3.16	*gltA*	Mal4_25960	Y	Y	Y
Aconitate hydratase A	4.2.1.3	*acnA*	Mal4_15300	Y	Y	Y
Isocitrate dehydrogenase [NADP]	1.1.1.42	*icd*	Mal4_26830	Y	Y	Y
2-Oxoglutarate dehydrogenase complex	1.2.4.2/2.3.1.61	*sucAB*	Mal4_26900/Mal4_26910	Y	Y	Y
Succinate-CoA ligase complex	6.2.1.5	*sucCD*	Mal4_08670/Mal4_08660	Y	Y	Y
Succinate dehydrogenase complex	1.3.5.1	*sdhABC*	Mal4_42560/ Mal4_42550/Mal4_42570	Y	Y	Y
Fumarate hydratase class I, an/aerobic	4.2.1.2	*fumAB*	N	N	N	n.a.
Fumarate hydratase class II	4.2.1.2	*fumC*	Mal4_09530	Y	Y	Y
Malate dehydrogenase	1.1.1.37	*mdh*	Mal4_25770	Y	Y	Y
Glyoxylate shunt						
Isocitrate lyase	4.1.3.1	*aceA*	N	N	N	n.a.
Malate synthase G	2.3.3.9	*glcB*	N	N	N	n.a.

*Genomic data from GenBank acc. no. NZ_FOQD00000000. Presence of a gene in strains used for comparison is indicated by ‘Y’ and absence is indicated by ‘N’

*n.a.* not available

Based on the physiological, morphological and phylogenetic analyses of strain Mal4^T^, we conclude that the characterised strain represents a novel species within the novel genus *Maioricimonas*. Thus, we propose the name *Maioricimonas rarisocia* gen. nov., sp. nov., represented by the type strain Mal4^T^.

### *Maioricimonas* gen. nov.

*Maioricimonas* (Ma.io.ri.ci’mo.nas. M.L. fem. n. *Maiorica* of Mallorca; L. fem. n. *monas* a unit, monad; N.L. fem. n. *Maioricimonas* a monad from Mallorca, Spain).

Cells have a Gram-negative cell envelope architecture and divide by polar budding. Cells are mesophilic, neutrophilic, aerobic and heterotrophic and present crateriform structures and matrix or fimbriae. The genus is part of the family *Planctomycetaceae*, order *Planctomycetales*, class *Planctomycetia*, phylum *Planctomycetes*. The type species of the genus is *Maioricimonas rarisocia.*

### *Maioricimonas rarisocia* sp. nov.

*Maioricimonas rarisocia* (ra.ri.so’ci.a. L. masc. adj. *rarus* few, infrequent; L. masc. adj. *socius* allied, united; N.L. fem. adj. *rarisocia*; corresponding to the characteristic of the cells to seldom form aggregates).

In addition to the features described for the genus, cells are pear-shaped (2.0 × 1.4 µm), form orange colonies and mostly occur as single cells. Temperature and pH optimum of the type strain are 31 °C and 7.5, respectively, however growth is observed over a range of 10–39 °C and pH 6.5–9.0. The type strain genome (accession number CP036275) and 16S rRNA gene (accession number MK559979) are available from GenBank. The genome of the type strain has a G+C content of 63.4% and a size of 7.7 Mb.

The type strain is Mal4^T^ (= DSM 100296^T^ = LMG 29133^T^, deposited as strain Malle4), isolated from marine sediments near the coast of S’Arenal in Palma de Mallorca, Mallorca Island, Spain.

## References

[CR1] Acehan D, Santarella-Mellwig R, Devos DP (2014). A bacterial tubulovesicular network. J Cell Sci.

[CR2] Bengtsson MM, Øvreås L (2010). Planctomycetes dominate biofilms on surfaces of the kelp *Laminaria hyperborea*. BMC Microbiol.

[CR3] Bengtsson MM, Sjøtun K, Lanzén A, Ovreås L (2012). Bacterial diversity in relation to secondary production and succession on surfaces of the kelp *Laminaria hyperborea*. ISME J.

[CR4] Boedeker C, Schüler M, Reintjes G (2017). Determining the bacterial cell biology of Planctomycetes. Nat Commun.

[CR5] Boersma AS, Kallscheuer N, Wiegand S (2019). *Alienimonas californiensis* gen. nov. sp. nov., a novel Planctomycete isolated from the kelp forest in Monterey Bay. Antonie Van Leeuwenhoek.

[CR6] Bondoso J, Albuquerque L, Nobre MF (2015). *Roseimaritima ulvae* gen. nov., sp. nov. and *Rubripirellula obstinata* gen. nov., sp. nov. two novel planctomycetes isolated from the epiphytic community of macroalgae. Syst Appl Microbiol.

[CR7] Bondoso J, Balagué V, Gasol JM, Lage OM (2014). Community composition of the Planctomycetes associated with different macroalgae. FEMS Microbiol Ecol.

[CR8] Bondoso J, Godoy-Vitorino F, Balagué V (2017). Epiphytic Planctomycetes communities associated with three main groups of macroalgae. FEMS Microbiol Ecol.

[CR9] Bondoso J, Harder J, Lage OM (2013). *rpoB* gene as a novel molecular marker to infer phylogeny in *Planctomycetales*. Antonie Van Leeuwenhoek.

[CR10] Castresana J (2000). Selection of conserved blocks from multiple alignments for their use in phylogenetic analysis. Mol Biol Evol.

[CR11] Dedysh SN, Henke P, Ivanova AA (2020). 100-year-old enigma solved: identification, genomic characterization and biogeography of the yet uncultured Planctomyces bekefii. Environ Microbiol.

[CR12] Devos DP (2014). PVC bacteria: variation of, but not exception to, the Gram-negative cell plan. Trends Microbiol.

[CR13] Devos DP (2014). Re-interpretation of the evidence for the PVC cell plan supports a Gram-negative origin. Antonie Van Leeuwenhoek.

[CR14] Devos DP, Ward NL (2014). Mind the PVCs. Environ Microbiol.

[CR15] Edgar RC (2004). MUSCLE: multiple sequence alignment with high accuracy and high throughput. Nucleic Acids Res.

[CR16] Faria M, Bordin N, Kizina J (2018). Planctomycetes attached to algal surfaces: insight into their genomes. Genomics.

[CR17] Fuerst JA, Webb RI (1991). Membrane-bounded nucleoid in the eubacterium *Gemmata obscuriglobus*. Proc Natl Acad Sci USA.

[CR18] Graça AP, Calisto R, Lage OM (2016). Planctomycetes as novel source of bioactive molecules. Front Microbiol.

[CR19] Guo M, Zhou Q, Zhou Y (2014). Genomic evolution of 11 type strains within family *Planctomycetaceae*. PLoS ONE.

[CR20] Jeske O, Jogler M, Petersen J (2013). From genome mining to phenotypic microarrays: Planctomycetes as source for novel bioactive molecules. Antonie Van Leeuwenhoek.

[CR21] Jeske O, Schüler M, Schumann P (2015). Planctomycetes do possess a peptidoglycan cell wall. Nat Commun.

[CR22] Jeske O, Surup F, Ketteniß M (2016). Developing techniques for the utilization of Planctomycetes as producers of bioactive molecules. Front Microbiol.

[CR23] Jogler C (2014). The bacterial “mitochondrium”. Mol Microbiol.

[CR24] Jogler C, Glöckner FO, Kolter R (2011). Characterization of *Planctomyces limnophilus* and development of genetic tools for its manipulation establish it as a model species for the phylum Planctomycetes. Appl Environ Microbiol.

[CR25] Jogler C, Waldmann J, Huang X (2012). Identification of proteins likely to be involved in morphogenesis, cell division, and signal transduction in Planctomycetes by comparative genomics. J Bacteriol.

[CR26] Kallscheuer N, Jogler M, Wiegand S (2019). *Rubinisphaera italica* sp. nov. isolated from a hydrothermal area in the Tyrrhenian Sea close to the volcanic island Panarea. Antonie Van Leeuwenhoek.

[CR27] Kallscheuer N, Moreira C, Airs R (2019). Pink- and orange-pigmented Planctomycetes produce saproxanthin-type carotenoids including a rare C_45_ carotenoid. Environ Microbiol Rep.

[CR28] Kallscheuer N, Wiegand S, Peeters SH (2019). Description of three bacterial strains belonging to the new genus *Novipirellula* gen. nov., reclassificiation of *Rhodopirellula rosea* and *Rhodopirellula caenicola* and readjustment of the genus threshold of the phylogenetic marker *rpoB* for *Planctomycetaceae*. Antonie Van Leeuwenhoek.

[CR29] Kohn T, Heuer A, Jogler M (2016). *Fuerstia marisgermanicae* gen. nov., sp. nov., an unusual member of the phylum *Planctomycetes* from the German Wadden Sea. Front Microbiol.

[CR30] Kohn T, Rast P, Wiegand S (2020). The microbiome of *Posidonia oceanica* seagrass leaves can be dominated by Planctomycetes. Front Microbiol.

[CR31] Kohn T, Wiegand S, Boedeker C (2020). *Planctopirus ephydatiae*, a novel Planctomycete isolated from a freshwater sponge. Syst Appl Mirobiol.

[CR32] König E, Schlesner H, Hirsch P (1984). Cell wall studies on budding bacteria of the *Planctomyces/Pasteuria* group and on a *Prosthecomicrobium* sp. Arch Microbiol.

[CR33] Konstantinidis KT, Rosselló-Móra R, Amann R (2017). Uncultivated microbes in need of their own taxonomy. ISME J.

[CR34] Kulichevskaya IS, Ivanova AA, Detkova EN (2015). *Planctomicrobium piriforme* gen. nov., sp. nov., a stalked planctomycete from a littoral wetland of a boreal lake. Int J Syst Evol Microbiol.

[CR35] Lachnit T, Fischer M, Künzel S (2013). Compounds associated with algal surfaces mediate epiphytic colonization of the marine macroalga *Fucus vesiculosus*. FEMS Microbiol Ecol.

[CR36] Lage OM, Bondoso J (2014). Planctomycetes and macroalgae, a striking association. Front Microbiol.

[CR37] Lechner M, Findeiß S, Steiner L (2011). Proteinortho: detection of (co-)orthologs in large-scale analysis. BMC Bioinformatics.

[CR38] Lee I, Ouk Kim Y, Park S-C, Chun J (2016). OrthoANI: an improved algorithm and software for calculating average nucleotide identity. Int J Syst Evol Microbiol.

[CR39] Liechti G, Kuru E, Packiam M (2016). Pathogenic *Chlamydia* lack a classical sacculus but synthesize a narrow, mid-cell peptidoglycan ring, regulated by MreB, for cell division. PLoS Pathog.

[CR40] Liechti GW, Kuru E, Hall E (2014). A new metabolic cell-wall labelling method reveals peptidoglycan in *Chlamydia* trachomatis. Nature.

[CR41] Lindsay M, Webb R, Fuerst J (1997). Pirellulosomes: a new type of membrane-bounded cell compartment in Planctomycete bacteria of the genus *Pirellula*. Microbiology.

[CR42] Lombard V, Golaconda Ramulu H, Drula E (2014). The carbohydrate-active enzymes database (CAZy) in 2013. Nucleic Acids Res.

[CR43] Lonhienne TGA, Sagulenko E, Webb RI (2010). Endocytosis-like protein uptake in the bacterium *Gemmata obscuriglobus*. Proc Natl Acad Sci USA.

[CR45] Mitchell AL, Attwood TK, Babbitt PC (2019). InterPro in 2019: improving coverage, classification and access to protein sequence annotations. Nucleic Acids Res.

[CR46] Neumann S, Wessels HJCT, Rijpstra WIC (2014). Isolation and characterization of a prokaryotic cell organelle from the anammox bacterium *Kuenenia stuttgartiensis*. Mol Microbiol.

[CR47] Pearson A, Budin M, Brocks JJ (2003). Phylogenetic and biochemical evidence for sterol synthesis in the bacterium *Gemmata obscuriglobus*. Proc Natl Acad Sci USA.

[CR48] Peeters SH, van Niftrik L (2019). Trending topics and open questions in anaerobic ammonium oxidation. Curr Opin Chem Biol.

[CR49] Pilhofer M, Aistleitner K, Biboy J (2013). Discovery of chlamydial peptidoglycan reveals bacteria with murein sacculi but without FtsZ. Nat Commun.

[CR50] Pilhofer M, Rappl K, Eckl C (2008). Characterization and evolution of cell division and cell wall synthesis genes in the bacterial phyla *Verrucomicrobia*, *Lentisphaerae*, *Chlamydiae*, and *Planctomycetes* and phylogenetic comparison with rRNA genes. J Bacteriol.

[CR51] Pruesse E, Peplies J, Glöckner FO (2012). SINA: accurate high-throughput multiple sequence alignment of ribosomal RNA genes. Bioinformatics.

[CR52] Qin Q-L, Xie B-B, Zhang X-Y (2014). A proposed genus boundary for the prokaryotes based on genomic insights. J Bacteriol.

[CR53] Rast P, Glöckner I, Boedeker C (2017). Three novel species with peptidoglycan cell walls form the new genus *Lacunisphaera* gen. nov. in the family *Opitutaceae* of the verrucomicrobial subdivision 4. Front Microbiol.

[CR54] Ravin NV, Rakitin AL, Ivanova AA (2018). Genome analysis of fimbriiglobus ruber SP5T, a planctomycete with confirmed chitinolytic capability. Appl Environ Microbiol.

[CR55] Rivas-Marín E, Canosa I, Devos DP (2016). Evolutionary cell biology of division mode in the bacterial *Planctomycetes*-*Verrucomicrobia*-*Chlamydiae* superphylum. Front Microbiol.

[CR56] Rivas-Marín E, Canosa I, Santero E, Devos DP (2016). Development of genetic tools for the manipulation of the planctomycetes. Front Microbiol.

[CR57] Rivas-Marin E, Stettner S, Gottshall EY (2019). Essentiality of sterol synthesis genes in the planctomycete bacterium *Gemmata obscuriglobus*. Nat Commun.

[CR58] Rivas-Marin E, Wiegand S, Kallscheuer N et al (2020) *Thalassoglobus polymorphus* sp. nov., a novel Planctomycete isolated close to a public beach of Mallorca Island. Antonie van Leeuwenhoek. 10.1007/s10482-020-01437-y 10.1007/s10482-020-01437-y PMC7716918 32583191

[CR59] Rodriguez-R LM, Konstantinidis KT (2016). The enveomics collection: a toolbox for specialized analyses of microbial genomes and metagenomes. PeerJ Preprints.

[CR60] Santarella-Mellwig R, Pruggnaller S, Roos N (2013). Three-dimensional reconstruction of bacteria with a complex endomembrane system. PLoS Biol.

[CR61] Scheuner C, Tindall BJ, Lu M (2014). Complete genome sequence of *Planctomyces brasiliensis* type strain (DSM 5305^T^), phylogenomic analysis and reclassification of Planctomycetes including the descriptions of *Gimesia* gen. nov., *Planctopirus* gen. nov. and *Rubinisphaera* gen. nov. and emended descriptions of the order *Planctomycetales* and the family *Planctomycetaceae*. Stand Genomic Sci.

[CR62] Sievers F, Wilm A, Dineen D (2011). Fast, scalable generation of high-quality protein multiple sequence alignments using Clustal Omega. Mol Syst Biol.

[CR63] Stamatakis A (2014). RAxML version 8: a tool for phylogenetic analysis and post-analysis of large phylogenies. Bioinformatics.

[CR64] Strous M, Kuenen JG, Jetten MSM (1999). Key physiology of anaerobic ammonium oxidation. Appl Environ Microbiol.

[CR65] van Teeseling MCF, Mesman RJ, Kuru E (2015). Anammox Planctomycetes have a peptidoglycan cell wall. Nat Commun.

[CR66] Vollmers J, Frentrup M, Rast P (2017). Untangling genomes of novel Planctomycetal and verrucomicrobial species from monterey bay kelp forest metagenomes by refined binning. Front Microbiol.

[CR67] Wagner M, Horn M (2006). The *Planctomycetes, Verrucomicrobia, Chlamydiae* and sister phyla comprise a superphylum with biotechnological and medical relevance. Curr Opin Biotechnol.

[CR68] Wiegand S, Jogler M, Boedeker C (2020). Cultivation and functional characterization of 79 planctomycetes uncovers their unique biology. Nat Microbiol.

[CR69] Wiegand S, Jogler M, Jogler C (2018). On the maverick Planctomycetes. FEMS Microbiol Rev.

[CR70] Yadav S, Vaddavalli R, Siripuram S (2018). Planctopirus hydrillae sp. nov., an antibiotic producing Planctomycete isolated from the aquatic plant Hydrilla and its whole genome shotgun sequence analysis. J Antibiot.

[CR71] Yarza P, Yilmaz P, Pruesse E (2014). Uniting the classification of cultured and uncultured bacteria and archaea using 16S rRNA gene sequences. Nat Rev Microbiol.

[CR72] Zhang H, Yohe T, Huang L (2018). dbCAN2: a meta server for automated carbohydrate-active enzyme annotation. Nucleic Acids Res.

